# Doxorubicin loaded Polymeric Nanoparticulate Delivery System to overcome drug resistance in osteosarcoma

**DOI:** 10.1186/1471-2407-9-399

**Published:** 2009-11-16

**Authors:** Michiro Susa, Arun K Iyer, Keinosuke Ryu, Francis J Hornicek, Henry Mankin, Mansoor M Amiji, Zhenfeng Duan

**Affiliations:** 1 Department of Orthopaedic Surgery, Massachusetts General Hospital, Boston, MA 02114, USA; 2 Sarcoma Biology Laboratory, Center for Sarcoma and Connective Tissue Oncology, Massachusetts General Hospital, Boston, MA 02114, USA; 3 Department of Pharmaceutical Sciences, School of Pharmacy, Northeastern University, Boston, MA 02115, USA; 4 Current address: Department of Radiology, Center for Molecular & Functional Imaging, University of California at San Francisco, San Francisco, CA 94107, USA

## Abstract

**Background:**

Drug resistance is a primary hindrance for the efficiency of chemotherapy against osteosarcoma. Although chemotherapy has improved the prognosis of osteosarcoma patients dramatically after introduction of neo-adjuvant therapy in the early 1980's, the outcome has since reached plateau at approximately 70% for 5 year survival. The remaining 30% of the patients eventually develop resistance to multiple types of chemotherapy. In order to overcome both the dose-limiting side effects of conventional chemotherapeutic agents and the therapeutic failure incurred from multidrug resistant (MDR) tumor cells, we explored the possibility of loading doxorubicin onto biocompatible, lipid-modified dextran-based polymeric nanoparticles and evaluated the efficacy.

**Methods:**

Doxorubicin was loaded onto a lipid-modified dextran based polymeric nano-system. The effect of various concentrations of doxorubicin alone or nanoparticle loaded doxorubicin on KHOS, KHOS_R2_, U-2OS, and U-2OS_R2 _cells was analyzed. Effects on drug retention, immunofluorescence, Pgp expression, and induction of apoptosis were also analyzed.

**Results:**

Dextran nanoparticles loaded with doxorubicin had a curative effect on multidrug resistant osteosarcoma cell lines by increasing the amount of drug accumulation in the nucleus via Pgp independent pathway. Nanoparticles loaded with doxorubicin also showed increased apoptosis in osteosarcoma cells as compared with doxorubicin alone.

**Conclusion:**

Lipid-modified dextran nanoparticles loaded with doxorubicin showed pronounced anti-proliferative effects against osteosarcoma cell lines. These findings may lead to new treatment options for MDR osteosarcoma.

## Background

Osteosarcoma is the most common primary malignant bone tumor in children and adolescents and accounts for approximately 60% of primary malignant bone tumors diagnosed in the first two decades of life [1]. Standard treatment for osteosarcoma is a combination of surgery and chemotherapy. The cure rate of patients with localized osteosarcoma ranges from 15 to 20% with surgery alone, but improves to approximately 70% when combined with chemotherapy [2]. Unfortunately, for the 40% of patients with progression of osteosarcoma after frontline therapy, further therapy with additional chemotherapy is palliative and toxic. It is estimated that less than 30% of patients with recurrent disease will be cured [2-4].

Cancer cells employ a host of different mechanisms to become resistant to one or more chemotherapeutic agents. It is known that multidrug resistance (MDR) can occur due to the following potential reasons: enhanced detoxification of the drugs through increased metabolism, decrease in drug uptake, a reaction with increased levels of intracellular nucleophiles, enhanced repair of the drug-induced damage to DNA [5], or through overexpression of membrane-bound drug transporter proteins, such as P-glycoprotein (Pgp, ABCB1), multidrug resistance-associated proteins (MRP1, ABCC1 and MRP2, ABCC2) and the breast cancer resistance protein (BCRP, ABCG2) [6]. The development and discovery of agents that reverse MDR with high efficiency and low toxicity has been the focus of extensive research [7-10]. Unfortunately, these compounds are often nonspecific and have low efficiency and/or high toxicity; as such, phase 3 clinical trials of these agents are largely disappointing [7,8,11-14]. Successful management of osteosarcoma would be greatly aided by novel agents that interfere with both intrinsic and acquired mechanisms of drug resistance mechanisms.

To overcome drug resistance and reduce the side effects during chemotherapy, nanotechnology holds promising potential, utilizing targeted drug delivery [15]. Varieties of nanoparticles are available [16]: polymeric nanoparticles, dendrimers, inorganic/metal nanoparticles, quantum dots, liposomes, micelles, and several other types of nano-assemblies. Polymeric nanoparticle based delivery systems offer a significant advantage over other nano-carrier platforms as there is tremendous versatility in choice of polymer matrices that can be used, which allows for the tailoring of nanoparticle properties to meet the specific needs they were intended to meet. Other advantages include ease in surface modification, greater encapsulation efficiency of the payload, payload protection, large surface area-to-volume ratio, and slow or fast polymer erosion for temporal control over the release of drugs [17].

Dextran is a polysaccharide mainly composed of 1,6-linked D-glucopyranose residues. There are several advantages of using dextran as a macromolecular carrier for chemotherapeutic applications because it is biocompatible and inert. Due to its biocompatibility and biodegradability, dextran and its derivatives have been widely used as blood substitutes and drug carriers [18]. Much work has been carried out to realize useful properties for dextran via various chemical modifications for specific applications, especially its use as a polymeric drug carrier [19,20]. Low molecular weight conjugates of dextran have the advantage of reduced side effects, however its therapeutic potential is not optimum, due to its relatively short plasma half-life [21]. Therefore, in this study, we have used high molecular weight dextran-lipid nanoparticles to encapsulate doxorubicin.

Although the drug resistance in osteosarcoma is well known, there is hardly any study that addresses the effect of drug-loaded nanoparticle formulation on drug-resistant osteosarcoma cells. In order to overcome both the dose-limiting side effects of conventional chemotherapeutic agents and the therapeutic failure incurred from MDR tumor cells, we have undertaken the rational design of biocompatible dextran based polymeric nanoparticles for the sustained delivery of doxorubicin.

## Methods

### Chemicals

Dextran (Mw~40 kDa), stearyl amine (99% pure), cystamine, pyridine, sodium periodate (NaIO_4_), sodium cyanoborohydride (NaCNBH_3_), and potassium sulfate (K_2_SO_4) _and azo-bis-isobutyronitrile (AIBN) were obtained from Sigma-Aldrich Chemical Co (St. Louis, MO). Dithiol-modified poly(ethylene glycol) (PEG-(SH)_2_, M.W. 2,000) was purchased from SunBio, Inc. (Seoul, South Korea). Anhydrous lithium chloride (LiCl) was from Fisher Scientific (Philadelphia, PA). Dehydrated dimethylformamide (DMF) and dimethylsulfoxide (DMSO) with molecular sieves was obtained from Acros Organics (Parsipanny, NJ). Acryloyl chloride, pyridine and other reagents and solvents were from Sigma-Aldrich and were used as received without further purification.

### Synthesis of lipid-modified dextran polymer

#### Synthesis of dextran acrylate

The synthesis of dextran acrylate was based on the procedure of Zhang et al. [22]. Briefly, a fixed amount of dextran (M.W. ~40 kDa, 2 g) was added to a LiCl/DMF (4% w/v, 50 ml) solvent mixture in a round bottom flask (200 ml). The temperature of the oil bath was raised from room temperature to 120°C over a period of 2 h. The resultant mixture became a homogeneous golden yellow solution. The solution was cooled to room temperature, and pyridine (500 μl) was added and stirred. The reaction mixture was cooled to 0°C using ice bath and varying amounts of acryloyl chloride (1-1.5 molar excess) was added drop wise using an addition funnel. The reaction was maintained at 0°C until complete addition of acryloyl chloride was done over a period of 1-2 h. The reaction was allowed to continue overnight. The dextran-acrylate obtained was precipitated in excess cold ethanol and washed three times with absolute ethanol. For confirmation of the formation of dextran acrylate, a small portion of the acrylate monomer was polymerized using 0.001% AIBN initiator in DMSO at 60°C for 24 hours, which resulted in formation of the acrylate polymer, confirming the reaction. Alternately, for lipid modification, the dextran-acrylate obtained in step 1 was directly used as the monomer for the next step, in a one-pot synthesis.

#### Lipid modification of dextran acrylate

A desired amount (200 mg) of dextran acrylate obtained from above step was dissolved in dry DMF and stirred in a 20 ml glass vial with varying amounts (5-10 mole %) of stearylamine and a catalyst (0.01 mole % AlCl_3_). The reaction mixture was heated to 40-50°C in an oil bath for 24 h. The product obtained (stearyl-modified dextran) was precipitated and washed in cold ethanol several times to purify the product. Finally, the lipid-modified dextran derivative was dissolved in small amount of deionized water and lyophilized to yield the pale yellow final product. The stearyl modification of dextran was confirmed by NMR spectroscopy and the % lipid modification was estimated to be 3-7 mole%.

### Synthesis of thiolated dextran

#### Oxidation of dextran

The dextran backbone was oxidized based on the procedure of Martwiset et al. [23]. Briefly, a desired amount of NaIO_4 _was dissolved in 60 ml of deionised water. The solution was added to another solution containing 4 g of dextran and 30 ml of de-ionised water. The reaction was stirred in the dark for 2 h at room temperature. At the end of the reaction, the solution was dialyzed using Spectrapor^® ^dialysis membranes (M.W. cutoff 12-14 kDa, Spectrum Labs, Rancho Dominguez, CA) extensively against de-ionized water (2 L) for 4 days with several water replacements. A powdery free-flowing sample was obtained after freeze-drying. Yield: 3.7 g (92.5%).

#### Thiol modification of dextran

A 500 mg portion of the oxidized dextran was dissolved in 50 ml pH 5.2 buffer containing K_2_SO_4 _and NaCNBH_3_. Then, 50 mg of cystamine was added and stirred at 40°C for 4 days. The product was subjected to extensive dialysis and then lyophilized to yield thiolated-dextran. The percent thiolation was quantified by Ellman's reagent [24]. The concentration of sulfohydryl groups in the purified thiolated dextran derivate was estimated to be ~14.2 μM/mg.

### Preparation of doxorubicin-containing dextran nanoparticles

A stock solution of 5 mg/ml PEG-(SH)_2_, stearylamine-modified dextran, and thiolated-dextran synthesized above were prepared in deionised water. A 10 mM (1 ml) stock solution of doxorubicin was used for the preparation of doxorubicin loaded nanoparticle. For preparation of mixture, 40 μl of dextran-stearyl amine was first added to a 35 μl (0.35 mM) solution of doxorubicin and mixed well using a vortex shaker. It was then incubated for 5 minutes. To this mixture, 40 μl dextran-thiol derivative was added and incubated for another 5 minutes. Finally, 40 μl of PEG-(SH)_2 _was added and incubated for another 15 minutes to form the hydrophilic shell of the nanoparticles. This method of sequential addition was used so that there is better interaction between the hydrophobic groups in doxorubicin and the lipid-modified dextran derivatives. Since the volume of the sample was small, it was gently vortexed and no stirring was used. The doxorubicin efficiency and loading in the nanoparticles was determined by measuring the absorbance of known amount of sample at 485 nm corresponding to λ max of doxorubicin and comparing the values to a standard curve obtained by using a stock solution of doxorubicin.

### Particle size and zeta potential measurements

The particle size and zeta potentials of doxorubicin loaded nanoparticles were performed with the Brookhaven Zeta PALS Instrument (Holtsville, NY). For light scattering experiments, the samples were measured at fixed angle of 90° at 25°C. The scattering intensity was adjusted in the range of 50-500 kcps by diluting the samples with deionised water. For zeta potentials, default parameters of dielectric constant, refractive index, and viscosity of water were used based on the electrophoretic mobility of the nanoparticles.

### Cell culture studies

Human osteosarcoma cell line U-2OS was obtained from the American Type Tissue Collection (Rockville, MD). Dr. Efstathios S. Gonos (National Hellenic research Foundation, Athens, Greece) kindly provided the human osteosarcoma cell line KHOS and the multidrug resistant cell lines KHOS_R2_, U-2OS_R2_. All cell lines were cultured in RPMI 1640 medium supplemented with 10% fetal bovine serum, 100 U/ml penicillin, and 100 ug/ml streptomycin (all obtained from Invitrogen, Carlsbad, CA). Cells were incubated at 37°C in 5% CO2-95% air atmosphere and passaged when near confluent monolayers were achieved using trypsin-EDTA solution. Resistant cell lines were continuously cultured in 0.1 uM doxorubicin. Doxorubicin was obtained as unused residual clinical material at the Massachusetts General Hospital (Bosston, MA).

#### Cell culture reagents

The Pgp1 monoclonal antibody C219 was purchased from Signet (Dedham, MA). The human β-actin monoclonal antibody and the MTT reagents were purchased from Sigma-Aldrich (St. Louis, MO).

### Quantitative and qualitative evaluation of doxorubicin uptake in cells

#### Flow cytometry

Cell suspensions of KHOS, KHOS_R2_, U-2OS, and U-2OS_R2 _incubated with doxorubicin with or without nanoparticle for 1 hour at 37°C were analyzed for the cellular fluorescence in a FACS Calibur flow cytometer (BD Biosciences, San Jose, CA) with data acquisition using CellQuest software. Doxorubicin is intrinsically fluorescent and can be excited with the 488 nm argon laser light [25]. The cells were washed and resuspended in PBS and fluorescence emission (above 530 nm) and forward angle light scatter were collected, amplified, and scaled to generate histograms. A minimum of 500,000 cells were analyzed for each histogram generated. Final doxorubicin concentration used was 10 μM.

#### Fluorescence microscopy

Cellular uptake studies were based on the procedure of Venne et al [26]. KHOS and KHOS_R2 _cells were seeded at densities of 5 × 10^5 ^cells/well in 6 well plates and incubated for 24 h to allow cell attachment. Following the incubation, either doxorubicin alone or nanoparticle loaded with doxorubicin was added to each well and were incubated for additional 3 hours. To counterstain nuclei, the cells were incubated with 1 μg/ml Hoechst 33342 (Invitrogen, Carlsbad, CA) for 1 minute. After incubation, the cells were washed and resuspended with PBS and were then visualized on a Nikon Eclipse Ti-U fluorescence microscope (Nikon Corp.) equipped with a SPOT RT digital camera (Diagnostic Instruments, Inc., Sterling Heights, MI). Fluorescence intensity and cellular localization was analyzed at a wavelength of 488 nm in triplicate different fields at random.

### Cytotoxicity assay

In vitro cytotoxity assays were performed by MTT assay as previously described. Briefly, 3 × 10^3 ^cells per well were plated in 96-well plate during this process. The culture medium used, RPMI 1640, contained increasing concentration of doxorubicin alone or nanoparticle loaded with doxorubicin. After culturing for 5 days, 10 μl of MTT (5 mg/ml in PBS) was added to each well and incubated for 3 hr. after dissolving the resulting formazan product with acid isopropanol, the absorbance (A490) was read on a SPECTRA max Microplate Spectrophotometer (Molecular Devices) at a wavelength of 490 nm. Experiment was performed in triplicate. The IC_50 _was defined as the compound or chemo drug concentration required decreasing the A_490 _to50% of the control value.

### Western Blot analysis

Pgp1 was analyzed in total cell lysates. Protein lysates from cells were generated through lysis with 1 × RIPA Lysis Buffer (Upstate Biotechnology, Charlottesville, VA). The concentration of the protein was determined by Protein Assay reagents (Bio-Rad, Hercules, CA) and a spectrophotometer (Beckman DU-640, Beckman Instruments, Inc., Columbia, MD). 25 μg of total protein was processed on Nu-Page 4-12% Bis-Tris Gel (Invitrogen) and transferred to pure nitrocellulose membrane (Bio-Rad Laboratories, Hecules, CA). Primary antibodies were incubated in Tris-buffered saline, pH7.4, with 0.1% Tween 20 overnight at 4°C. Signal was generated through incubation with horseradish peroxidase-conjugated secondary antibodies (Bio-Rad, Hercules, CA) incubated in Tris-buffered saline, pH 7.4, with 5% nonfat milk and 0.1% Tween 20 at 1: 2000 dilution for 1 h at room temperature. Positive immunoreactions were detected by using Super Signal^® ^West Pico Chemiluminescent Substrate (Pierce, Rockford, IL).

### Cellular apoptosis assay

Whole-cell lysates were immunoblotted with specific antibodies to PARP (Cell Signaling Technology) and its cleavage products. Positive immunoreactions were detected by using Super Signal^® ^West Pico Chemiluminescent Substrate. Bands were semiquantified by reverse image scanning densitometry with PhotoShop 7.0 (Adobe, San Jose, CA). An area of the gel image that was devoid of signal was assigned to be the background value. Then, each band of the protein representing cleaved PARP from KHOS or KHOS_R2 _treated with either doxorubicin alone or nanoparticle loaded with doxorubicin at various concentrations were analyzed for the density beyond background level. To ensure that the loading of the protein was equal and differences were not being observed because of one specimen having more protein than another, the band corresponding to β-actin was determined for each protein. The density of the protein band was normalized to the β-actin band for the protein and the ratio of the cleaved PARP was normalized by dividing by the ratio of the actin corresponding to each cleaved PARP. As a second parameter of apoptotic cell death, we measured caspase-3/7 activity in KHOS and KHOS_R2 _after treatment with either doxorubicin alone or nanoparticle loaded with doxorubicin by using Apo-ONE Homogenous caspase-3/7 system according to the manufacturer's instructions (Promega, Madison, WI). The intensity of the emitted fluorescence was determined at a wavelength of 521 nm with the use of a SPECTRAmax^® ^Microplate Spectrofluorometer (Molecular Devices).

### Statistical analysis

Values shown are representative of triplicate determinations in two or more experiments. Student's t-test was used to compare the differences between groups (GraphPad PRISM^® ^4 software, GraphPad Software, San Diego, CA). Results are given as mean ± SD and results with p < 0.05 were considered statistically significant.

## Results

### Lipid-modified dextran nanoparticles for intracellular doxorubicin delivery

**Figure 1:**
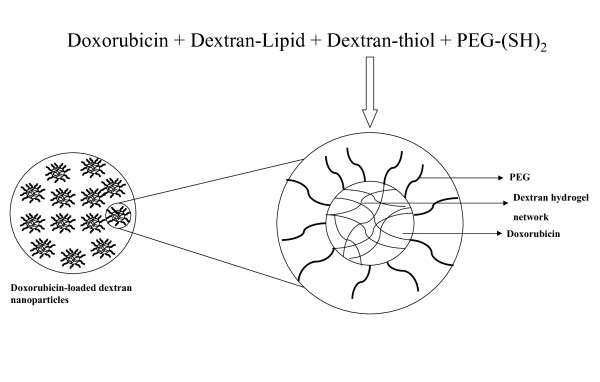
**Pictorial representation ofdoxorubicin loaded stearylamine-dextran modified nanoparticles**. Thelipid - modified dextran forms hydrophobic association withdoxorubicin and the thiol modified dextran interacts with thethiolated\poly (ethylene glycol) (PEG), forming a core shellstructure, which is further stabilized by the PEG chains.

Doxorubicin was incubated with the lipid-modified dextran derivative in deionized water at RT to form nanoparticles by self-assembled hydrophobic interactions. Further a thiolated-dextran derivative and PEGylated-thiol derivative was sequentially mixed with the doxorubicin containing lipid-modifed dextran derivated to enhance the binding efficiency of PEG chains to the dextran hydrogel. This method in fact helped in formation of stable nanoparticles with good doxorubicin loading (Figure 1). The mean particle size of the doxorubicin-loaded nanoparticles as determined by dynamic light scattering (DLS) measurement was 112.4 ± 4.2 nm and the zeta potential was almost neutral (+1.19 ± 0.82 mV). The particles were stable at room temperature and there was not much change in the particle size on storage (for 1 week at 4°C).

### Enhancement of intracellular doxorubicin accumulation with nanoparticle delivery

**Figure 2:**
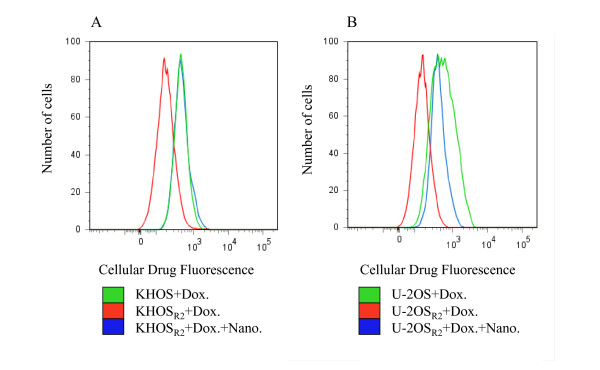
**Fluorescence of KHOS, KHOS_R2 _(A), U-2OS, and U-2OS_R2 _(B) after treatment with doxorubicin aloneor nanoparticle loaded with doxorubicin was analyzed by flow cytometry**. The effect of nanoparticle was potent enough that thefluorescence of MDR osteosarcoma cells was comparable to that of drugsensitive variants.

Lipid-modified dextran nanoparticle caused a significant increase in the retention of doxorubicin in both KHOS_R2 _(Figure 2A) and U-2OS_R2 _(Figure 2B) when examined through flow cytometry. It was potent enough that the fluorescence of drug resistant cells treated with doxorubicin loaded nanoparticles was comparable to that of drug sensitive cells treated with doxorubicin alone.

**Figure 3:**
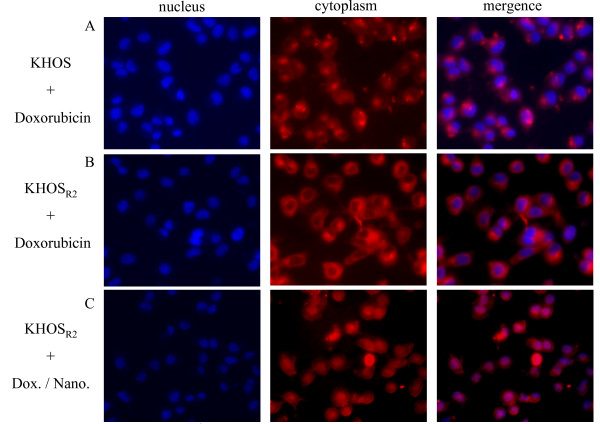
**Subcellular distribution of doxorubicin in drug sensitive and resistant osteosarcoma cell lines was analyzed under fluorescence microscope (A-C)**. A prominent increase in fluorescence was observedin the nucleus when multidrug resistant cells were treated withdoxorubicin loaded nanoparticles.

Using fluorescent microscopy, subcellular distribution of doxorubicin in KHOS and KHOS_R2 _was analyzed. After 3 hour incubation with free doxorubicin in drug resistant osteosarcoma cells, the drug was primarily concentrated in the cytoplasm, and a very low level of fluorescence was observed in the nucleus (Figure 3B). When doxorubicin was administered with the nanoparticle to drug resistant cell line, a prominent increase in fluorescence was observed in the nucleus, whereas the fluorescence of the cytoplasm remained virtually unaffected (Figure 3C). This subcellular distribution mimicked that of the drug sensitive variant when treated with doxorubicin (Figure 3A).

### Evaluation of anti-proliferative effects in wild-type and resistance cells

**Figure 4:**
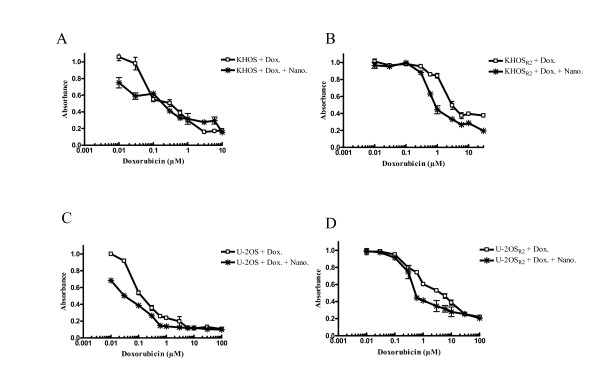
**The effect of doxorubicin alone or nanoparticle loaded with doxorubicin on KHOS, KHOS_R2_, U-2OS, andU-2OS_R2 _was analyzed (A-D)**. Varying concentrations ofdoxorubicin was added and consequently cultured for 5 days. Nanoparticle loaded with doxorubicin showed increasedanti-proliferative activity in both drug sensitive and resistantosteosarcoma cell lines in a dose dependent manner. Growth inhibition was assessed by MTT as described under Methods. The experiment wasrepeated four times in triplicate.

Dextran nanoparticle was non-cytotoxic by itself at a dose utilized in this study (Additional file 1). However, we found that nanoparticles loaded with doxorubicin showed an increased amount of anti-proliferative activity in both drug sensitive and resistant osteosarcoma cell lines in a dose dependent manner (Figure 4A-D). Nanoparticles loaded with doxorubicin showed a 10 fold higher activity compared to doxorubicin alone against U-2OS (IC_50 _0.03 μM to 0.3 μM, Figure 4C), 5 fold higher activity against KHOS_R2 _(IC_50 _0.6 μM to IC_50 _3 μM, Figure 4B), and 20 fold higher activity against U-2OS_R2 _(IC_50 _0.3 μM to 6 μM, Figure 4D).

**Figure 5:**
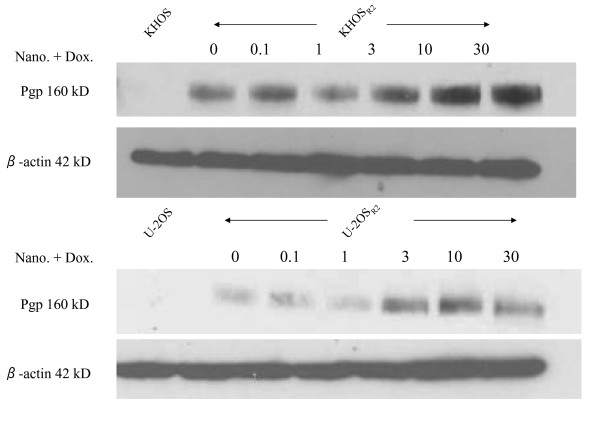
**Western blot assay was performed to assess the effect of doxorubicin loaded nanoparticle on expression of Pgp**. The expression of Pgp gradually increased along with the increase in the concentration of doxorubicin on both MDR osteosarcoma cell lines.

To estimate the effect of nanoparticle on Pgp expression, we utilized the Western blot assay. Pgp was not expressed in drug sensitive KHOS and U-2OS, however, Pgp was overexpressed in the two drug resistant cell lines KHOS_R2 _and U-2OS_R2 _(Figure 5). The nanoparticle did not suppress the expression of Pgp, but in contrast, the expression of Pgp gradually increased along with the increase in the concentration of doxorubicin. These effects were observed in two different drug resistant osteosarcoma cell lines (Figure 5).

### Induction of cellular apoptosis with doxorubicin nanoparticles

**Figure 6:**
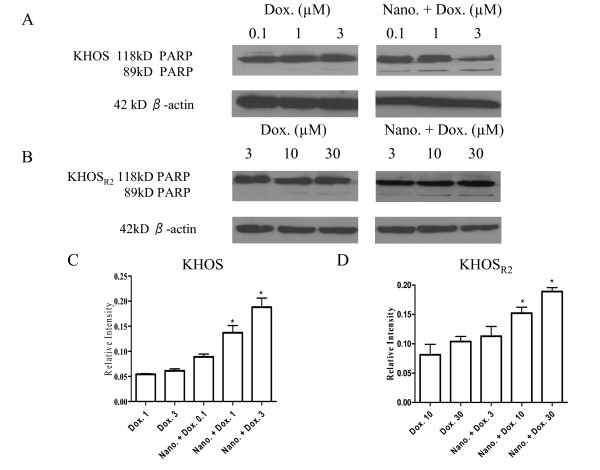
**Cleavage of PARP was detected using western blot assay**. Significant increase in apoptosis was observed for drug sensitive (A) and multidrug resistant osteosarcoma cells (B) when they were treated with doxorubicin loaded nanoparticle. Bar graphs represent the result of semiquantification by reverse image scanning densitometry with PhotoShop 7.0 (C, D). *P *values are shown as follows: *P < 0.05.

**Figure 7:**
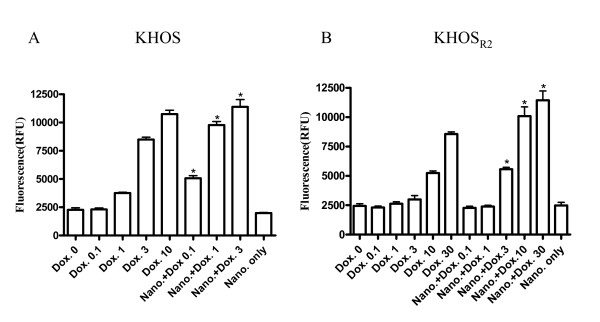
**Caspase-3/7 activity was measured as a second parameter of apoptotic cell death**. KHOS (A) and KHOS_R2 _(B) showed significant increase in apoptosis when they were treated with doxorubicin loaded nanoparticle. The experiment was repeated three times in triplicate. *P *values are shown as follows: *P < 0.05.

To assess the efficacy of nanoparticles loaded with doxorubicin to induce apoptosis on KHOS and KHOS_R2_, cleavage of PARP was detected using western blot assay. The dextran nanoparticle itself did not cause cleavage of PARP at a dose utilized in this study (Additional file 2). Although higher concentration of doxorubicin alone induced apoptosis, nanoparticles loaded with doxorubicin exhibited a much higher apoptosis induction rate in both drug sensitive (Figure 6A, C) and resistant cell lines (Figure 6B, D). In addition, caspase-3/7 activity was significantly increased when KHOS and KHOS_R2 _were treated with nanoparticles loaded with doxorubicin (Figure 7A, B).

## Discussion

Osteosarcoma is the most common primary malignant bone tumor among children and adolescents, and although the prognosis has improved dramatically in the last 30 years, a plateau in the survival curve has been reached at approximately 70% for 5-year survival [1,2]. Many studies are undergoing at present including several phase 3 studies to improve the prognosis of patients, but have not resulted in drastic improvements. In this report, a new nanoparticle formulation of co-encapsultaed doxorubicin with high potency has been devised and its efficacy against drug resistant osteosarcoma cell lines has been examined.

In the clinical setting, we have always faced the problem of dose limitation, defined as the systemic toxicity of conventional chemotherapeutics, an issue that can potentiate the MDR tumor phenotype. A narrow therapeutic index limits the dose that can be applied at any given single treatment, causing an uneven dose of treatment and resulting in some population of tumor cells experiencing low doses of the applied chemotherapeutic agent. This essentially promotes the cells for the development of MDR. Our study demonstrated that nanoparticles loaded with low concentrations of doxorubicin that will be free of cellular damage showed stronger and enhanced antiproliferative activity against both drug sensitive and resistant osteosarcoma cell lines.

In order to develop a biocompatible and safe nanoparticulate system for the delivery of doxorubicin, we chose to synthesize hydrophobically-modified dextran derivates, which can encapsulate doxorubicn by hydrophobic interactions, similar to the design reported for self-assembled nanosystems [27]. Further a thiolated-dextran derivative and PEGylated-thiol derivative was sequentially mixed with the lipid-modified dextran derivated to enhance the binding effeciency of PEG chains to the dextran hydrogel. PEGylated liposomes and nanoparticles can impart stealth character to the nanoparticles, increase the plasma residence time, protect the drug payload from degradation during circulation and also passively target tumor tissues due to the leakiness of the neovasculature by the enhanced permeability and retention (EPR) effect [28].

One of the main features of nanoparticles used for drug delivery is their preferable accumulation in solid tumors because of the EPR effect [29]. It has been shown that after administration of nanoparticles without drugs, the EPR effect in solid tumors primarily arose from differences in the clearance rate between solid tumor and normal tissues, after an initial penetration of the polymers into these tissues [30]. This is due to the increased tumor vascular permeability and the poor lymphatic drainage of the tumor tissue. It has been reported that nanoparticle bound drugs is 45-250 times higher in the site of tumors when compared to other organs such as the liver, kidney, lung, spleen, or heart [31]. The actual accumulation inside cells and preservation of drug antitumor activity during its tissue and subcellular trafficking are also important determinants of the efficacy of an anticancer agent. For anthracyclines in MDR cells, it has been reported that, in addition to a decrease in the drug accumulation, the intracellular distribution of the drug is changed in human myeloma [32], myeloid [33], lung tumor cells [34], ovarian carcinoma cells [35], and in epidermoid carcinoma cells [36]. Anthracycline fluorescence was found mainly in the nucleus inside sensitive cells and mainly in the cytoplasm in cells with a relatively high level of resistance [32-34,36]. The question was raised whether this was also applicable to osteosarcoma cell lines. In this study, cellular uptake and intracellular distribution of doxorubicin were examined under fluorescence microscopy. Under immunofluorescence microscope, doxorubicin was primarily accumulated in the nucleus of drug sensitive cells, and in the cytoplasm of drug resistant cells. When doxorubicin was loaded onto nanoparticles and applied to the drug resistant cells, drug distribution mimicked that of sensitivecells. These observations indicate that the nanoparticle formulation is capable of delivering doxorubicin to the nucleus even for the MDR strains. One of the proposed mechanisms that allow nanoparticles to be effective against the MDR cells is their ability to overcome drug efflux pumps such as Pgp in the cell membrane. In order to assess the effect of doxorubicin loaded nanoparticles on the expression of Pgp, we undertook western blot analysis. Several studies report that drug loaded nanopaticles inhibit *MDR1 *genes encoding drug efflux pumps, at the same time downregulating genes responsible for the drug detoxification and nonspecific resistance [37,38]. Interestingly, after 48 hours of incubation with doxorubicin loaded nanoparticle, Pgp was upregulated in the drug resistant cell lines in a dose dependent manner. Recently, several groups have demonstrated that basal expression of *MDR1 *is mechanistically controlled at the chromatin level and that the promoter methylation is a dominant epigenetic regulator of *MDR1 *transcription [39-42]. It seems that the nanoparticle delivery system caused increased uptake of doxorubicin and distribution in the nucleus that led to specific epigenetic modifications within the *MDR1 *region concomitant with *MDR1 *upregulation.

Several *in vitro *studies have suggested that nanoparticle-drug conjugates induce stronger activation of apoptosis-signaling pathways compared with free doxorubicin [37,43]. In contrast, others indicate that cell death induced by the same conjugates occurs primarily bynecrosis [44].

In our study, nanoparticles loaded with doxorubicin showed increased apoptosis compared to free doxorubicin, supporting the former results. An additional topic of current investigation is the co-administration of conventional chemotherapy with an efflux pump inhibitor combined with nanoparticles. There have been reports of sequential or concurrent administration of separate Pgp inhibitors and anticancer drugs [45], but this method cannot guarantee the co-action of intended drugs in the same cancer cells due to their different pharmacokinetics and tissue disposition. Introduction of use of nanoparticle based systems could improve the drug uptake and cytotoxity due to the coexistence of the chemosensitizer, anticancer drugs, and perhaps short nucleic acids such as MDR1siRNA in the same cell, but the precise mechanism of interaction warrants further investigation.

## Conclusion

Doxorubicin loaded dextran nanoparticle has resulted in a new cancer chemotherapeutic formulation. The indiscriminate ability of the formulation allows it to obliterate both wild type and the MDR osteosarcoma cells in vitro by drug accumulation in the nucleus and increased levels of apoptosis.

## Abbreviations

MDR: multidrug resistance; Pgp: P-glycoprotein; MRP: multidrug resistance-associated protein; BCRP: breast cancer resistance protein; NaIO_4_: sodium periodate; NaCNBH_3_: sodium cyanoborohydride; K_2_SO_4_: potassium sulfate; AIBN: azo-bis-isobutyronitrile; PEG-(SH)_2_: dithiol-modified poly(ethylene glycol); LiCl: lithium chloride; DMF: dehydrated dimethylformamide; DMSO: dimethylsulfoxide; DLS: dynamic light scattering; EPR: enhanced permeability and retention.

## Competing interests

The authors declare that they have no competing interests.

## Authors' contributions

MS carried out all the studies, analyzed the data, and wrote the first draft of the paper. AKI carried out the synthesis, characterization of the polymeric nanosystems, provided guidance with the study and assisted with the manuscript draft. KR helped in carrying out the experiments. FJH and HM provided experimental reagents and guided the team in the analysis of results and discussions. MMA provided experimental reagents and assisted with the manuscript draft. ZD is the principal investigator who led the research effort, provided guidance with the studies, assisted in data analysis and interpretation, and edited the manuscript. All authors read and approved the final manuscript.

## Pre-publication history

The pre-publication history for this paper can be accessed here:

http://www.biomedcentral.com/1471-2407/9/399/prepub

## Supplementary Material

Additional file 1 **The effect of nanoparticle on KHOS and KHOS_R2 _was analyzed**. The dextran nanoparticle was non-cytotoxic by itself at a dose utilized in this study. The experiment was repeated four times in triplicate. Click here for file

Additional file 2 **The effect of nanoparticle on cleavage of PARP was analyzed using western blot assay**. The dextran nanoparticle itself did not cause cleavage of PARP on KHOS or KHOS_R2 _at a dose utilized in this study. Click here for file
