# Supplementary figures and images for: Doxorubicin loaded Polymeric Nanoparticulate Delivery System to overcome drug resistance in osteosarcoma
Source: BMC Cancer. 2009 Nov 16;9:399. doi: 10.1186/1471-2407-9-399 (PMC2788581; doi:10.1186/1471-2407-9-399)

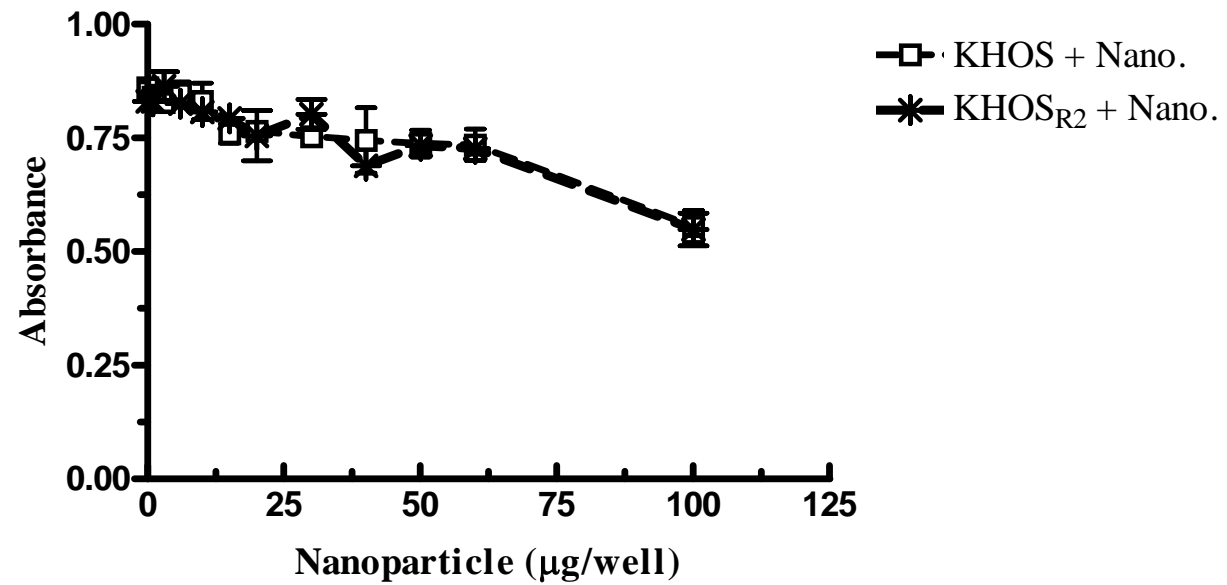

The effect of nanoparticle on KHOS and KHOS<sub>R2</sub> was analyzed.

Supplement: Additional file 1 — The effect of nanoparticle on KHOS and KHOSR2 was analyzed. The dextran nanoparticle was non-cytotoxic by itself at a dose utilized in this study. The experiment was repeated four times in triplicate. [file 1471-2407-9-399-S1.PDF]

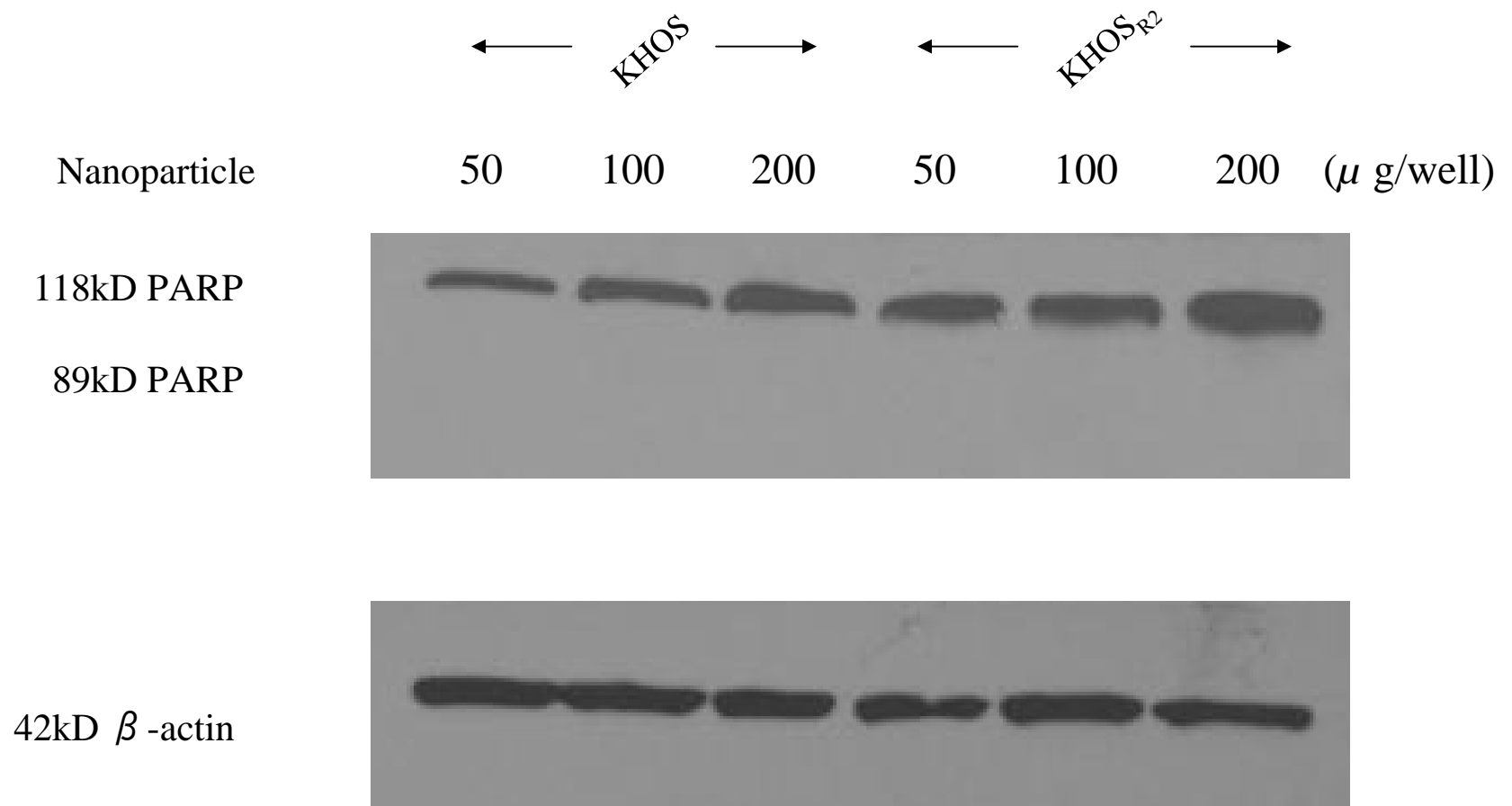

**The effect of nanoparticle on cleavage of PARP was analyzed using western blot assay.**

Supplement: Additional file 2 — The effect of nanoparticle on cleavage of PARP was analyzed using western blot assay. The dextran nanoparticle itself did not cause cleavage of PARP on KHOS or KHOSR2 at a dose utilized in this study. [file 1471-2407-9-399-S2.PDF]
